# Examining the validity and consistency of the Adult Eating Behaviour Questionnaire-Español (AEBQ-Esp) and its relationship to BMI in a Mexican population

**DOI:** 10.1007/s40519-021-01201-9

**Published:** 2021-05-08

**Authors:** Claudia Hunot-Alexander, Laura Patricia Arellano-Gómez, Andrea D. Smith, Martha Kaufer-Horwitz, Edgar M. Vásquez-Garibay, Enrique Romero-Velarde, Alison Fildes, Helen Croker, Clare H. Llewellyn, Rebecca J. Beeken

**Affiliations:** 1 Instituto de Nutrición Humana, Centro Universitario de Ciencias de la Salud, Universidad de Guadalajara, Edificio anexo al Nuevo Hospital Civil de Guadalajara “Dr. Juan I. Menchaca”, 3ª piso ala norte. Salvador Quevedo y Zubieta No. 750, C.P. 44340 Guadalajara, Jalisco Mexico; 2 Licenciatura en Nutrición y Ciencias de los Alimentos, Departamento de Psicología, Educación y Salud (DPES), Instituto Tecnológico de Estudios Superiores de Occidente (ITESO), Periférico Sur Manuel Gómez Morín #8585, C.P. 45604 Tlaquepaque, Jalisco Mexico; 3 Department of Behavioural Science and Health, University College London, London, WC1E 6BT UK; 4 Clínica de Obesidad y Trastornos de la Conducta Alimentaria, Departamento de Endocrinología y Metabolismo, Instituto Nacional de Ciencias Médicas y Nutrición, Salvador Zubirán, Avenida Vasco de Quiroga No.15, Colonia Belisario Domínguez Sección XVI, Delegación Tlalpan, C.P.14080 Ciudad de México, Mexico; 5 School of Psychology, University of Leeds, Leeds, LS2 9JT UK; 6 Leeds Institute of Health Sciences, University of Leeds, Leeds, LS2 9NL UK

**Keywords:** Appetite, Appetitive traits, Adults, Eating behaviour, Weight, Obesity, Behavioural susceptibility theory

## Abstract

**Purpose:**

Appetitive traits in adults and their associations with weight can be measured using the Adult Eating Behaviour Questionnaire (AEBQ). The aim of this study was to confirm the factor structure of the Spanish AEBQ (AEBQ-Esp) in a Mexican sample and explore associations between the eight traits with body mass index (BMI).

**Method:**

A sample of 1023 adults, mean age of 36.8 ± 12.8 years, was recruited from Guadalajara, Mexico. Researchers weighed and measured participants, and they completed the AEBQ-Esp either online or in paper format and reported sociodemographic data. To test two alternative factor structures (eight factors including Hunger; seven factors excluding Hunger), confirmatory factor analysis (CFA) was used. Internal reliability was assessed using Cronbach’s alpha; test–retest reliability was assessed using intra-class correlation coefficients. Multivariate linear regressions were used to test for associations between the AEBQ subscales and BMI, adjusted for age, sex, format of AEBQ responses, education, marital and employment status.

**Results:**

A seven-factor structure was the best model fit using CFA, excluding the Hunger subscale but similar to the original AEBQ. Internal reliability was good for all subscales (Cronbach’s *α* = 0.70–0.86), and the intra-class correlation coefficient (0.70–0.91) reflected good test–retest reliability. In the fully adjusted models, Satiety Responsiveness [*β* = − 0.61; (− 1.01, − 0.21)] and Slowness in Eating [*β* = − 0.70; (− 1.01, − 0.39)] were negatively associated with BMI, and Emotional Over-Eating [*β* = 0.94; (0.62, 1.27)] was positively associated with BMI.

**Conclusions:**

The AEBQ-Esp (excluding Hunger) appears to be a valid and reliable psychometric questionnaire for measuring appetitive traits in a Mexican Spanish-speaking population. Some traits appear to be associated with BMI in adulthood and warrant further exploration.

**Level of evidence:**

Level III evidence obtained from well-designed cohort or case–control analytic studies. Although this was just an observational study, it was well designed and provided new evidence.

**Supplementary Information:**

The online version contains supplementary material available at 10.1007/s40519-021-01201-9.

## Background

The growing prevalence of obesity worldwide has increased the need to understand better the variation in individual susceptibility to weight gain [1]. According to the Behavioural Susceptibility Theory (BST), appetitive traits such as food responsiveness (wanting to eat in response to the sight, smell or taste of palatable food) and satiety responsiveness (fullness threshold) are thought to play a role in an individual’s susceptibility to obesity [2, 3]. Appetitive traits are behavioural tendencies towards food and eating occasions. Inter-individual variation in appetitive traits such as Food Responsiveness and Satiety Responsiveness has been observed as early as three months of age [4]. This is evidence that some appetitive traits can be observed early in life and track into adolescence [2]. In children, appetitive traits are measured using the Child Eating Behaviour Questionnaire (CEBQ) [5]. Some of the traits captured by the CEBQ are thought to be, in part, genetically influenced. For example, twin studies have reported moderate to high heritability estimates for CEBQ-measured appetitive traits [6–8].

The CEBQ [5], is a consistent 35-item parent report questionnaire. It has been translated into many languages, including Spanish, Portuguese, French and Dutch [9–12], and its validity (the extent to which a questionnaire measures what it is supposed to measure) has been demonstrated in different countries. Studies using the CEBQ with children have consistently found it has a similar factor structure. Additionally, a number of the traits measured by this questionnaire have consistently been associated with variation in adiposity [5, 9, 13], both cross-sectionally and over time [14].

The CEBQ has recently been adapted into a self-report questionnaire, the Adult Eating Behaviour Questionnaire (AEBQ). The AEBQ was developed and validity examined in a British population and it measures eight traits: four ‘food approach’ or ‘eating onset’ traits: Hunger, Food Responsiveness, Emotional Over-Eating and Enjoyment of Food; and four ‘food avoidance’ or ‘eating off-set’ traits: Satiety Responsiveness, Emotional Under-Eating, Food Fussiness and Slowness in Eating. In line with observations in children using the CEBQ, negative associations between the ‘food avoidance’ traits (except Food Fussiness) and body mass index (BMI), and positive associations between the ‘food approach’ traits (except Hunger) and BMI were found in this adult sample [15].

Other commonly used psychometric measures of appetite include the ‘Three Factor Eating Questionnaire’ (TFEQ) [16] and the ‘Dutch Eating Behaviour Questionnaire’ (DEBQ) [17]. Studies using these measures have also found some evidence for associations between appetite and weight. For example, a number of studies utilizing the DEBQ have demonstrated associations between weight and both external eating (which correlates with Food Responsiveness), and Emotional Eating in adults [16–19].

Recently the validity of the AEBQ was examined for use in a sample of 998 Australian young adults [20]. Findings supported the AEBQ as a reliable and valid measure of appetitive traits in adults, although a better model fit was found for a seven-factor model which excluded Hunger. Mallan et al. [20], also found positive associations between BMI and Emotional Over-Eating (*r* = 0.14; *p* < 0.01), and negative associations between BMI and Satiety Responsiveness (*r* = − 0.17; *p* < 0.01), Food Fussiness (*r* = − 0.15; *p* < 0.01) and Slowness in Eating (*r* = − 0.16; *p* < 0.01). The authors hypothesized that the null associations between BMI and Food Responsiveness and Enjoyment of Food could reflect adults successfully trying to control their weight by exerting self-regulation of food intake, which would reduce the expression of these traits. Similar results have also been found in a Chinese [21] and Bulgarian [22] populations, whereas in a small sample of 50 female university students between the ages of 20–30 years old in Chile no associations between any of the traits and BMI were found [23].

To date the construct validity of the AEBQ has not been examined among non-English speaking samples. Examining a Spanish version of the AEBQ is important, given that Spanish is the official language spoken in 21 countries and the majority of Latin America. Validating the AEBQ in a Mexican sample, may be particularly valuable since Mexico is a transitional country in which obesity is a considerable public health concern [24, 25]. Improving our understanding of individual traits associated with higher BMIs in such a setting could inform the development of strategies that may help to manage these ‘higher-risk’ tendencies. For example, providing tailored behavioural advice to help individuals recognize and manage low Satiety Responsiveness in a food permissive environment may be helpful if associations between Satiety Responsiveness and BMI hold in this population [26]. Also, the very different food environment and culture of Mexico in comparison to the UK and Australia, provide a valuable opportunity for establishing the validity of the BST in a transitional country with high obesity rates. If the central hypothesis of the BST holds true in a country such as Mexico, which has a highly obesogenic and food permissive environment, then individuals with high ‘food approach’ and low ‘food avoidance’ traits would be at increased risk for sustained positive energy balance.

It is also important to investigate the role of weight management efforts on the relationships between weight and appetitive traits [27, 28]. Weight management may limit the extent to which appetitive traits are observed or heighten an individual’s awareness of certain traits. Identification of certain traits as problematic may also be associated with weight management and BMI and could make weight management harder or easier for some individuals [26]

The inclusion of the Hunger subscale in the AEBQ remains a concern, given that Hunger may be interpreted to be an appetite ‘state’, rather than a ‘trait’ [15, 29]. The AEBQ has recently been validated in a sample of British adolescents [20, 30]. Both the Australian and the adolescent validation studies suggested that the best model fit for the AEBQ (based on CFA) did not include the Hunger subscale and suggested it could be excluded in future studies. In contrast, the Chinese examination of the AEBQs validity [5] suggested that the original eight-factor model (including the Hunger subscale) had a better model fit compared to a seven-factor structure which included a joint Hunger-Food Responsiveness factor. However, they did not carry out a CFA excluding the Hunger subscale. Going forward, it will remain important to explore in more detail whether this subscale should be retained in studies using the AEBQ. The remaining factors, which were derived from the original CEBQ, are not expected to change given studies using the CEBQ with children have consistently found similar factor structures [9, 12, 31].

Therefore, the aims of this study were to: (1) confirm the factor structure of the Spanish version of the AEBQ, the AEBQ-Esp (Español); (2) determine the internal and test–retest reliability (external reliability) of the AEBQ-Esp, and (3) explore associations between the appetitive traits measured using the AEBQ-Esp with BMI in an adult population in Mexico, taking into account if participants were actively trying to lose weight or not.

## Methods

### Translation and Think-Aloud methodology

A researcher (author CH) who is bilingual in both English and native Mexican Spanish performed the forward translation of the AEBQ into Spanish. A bilingual panel of experts, consisting of four bilingual participants and one certified translator, then checked the item translations that would be most suited to a Mexican Spanish speaking population (Supplementary material 1). To improve face (content) validity of the questionnaire, the translation of the AEBQ was verified by structured cognitive testing [32], using a qualitative approach known as Think-Aloud interviews to gain insight into participants’ understanding of questionnaire items [33]. These interviews were conducted in adults of different genders, age and education levels, using paraphrasing techniques and probes, whilst asking participants to read out loud the questionnaire. Think aloud interviews were recorded using a digital voice recorder. Finally backward translation of the AEBQ into English was carried out to check the AEBQ had been translated appropriately.

### Participants and procedure

Data from an opportunity sample of adults living in Guadalajara, in the central western region of Mexico were collected from March to June 2017. Researchers invited participants to take part in the study, by approaching them directly, at the dental clinic, which is part of the Health Sciences University Center of the University of Guadalajara. This procedure was also used with university administrative staff and students. The researchers collecting the data also invited their family members and friends of different age groups, to complete the questionnaire. Participants who responded online were asked if they would like to respond to a second questionnaire 2 weeks later by leaving their email, and those who accepted were contacted to take part in the test–retest. The main exclusion criteria were participants whose first language was not Spanish, thus no indigenous populations were sampled. All participants were weighed and measured by researchers, and completed a questionnaire containing questions about socio-demographics and the AEBQ-Esp. Participants were free to choose whether to complete the questionnaire online through Survey Monkey, or as a paper version. A paper version was offered alongside the online version to try and obtain a larger and more demographically varied sample of participants, including those who had little access to the internet. In the first stages of the study, brief consistency checks were undertaken to check for differences in the responses obtained online compared to those obtained by paper and pencil. Irrespective of data collection medium, data were pooled and we subsequently controlled for these differences.

Ethical approval (number CI-1217) was obtained from the Comité de Bioética e Investigación del Centro Universitario de Ciencias de la Salud, Universidad de Guadalajara. Informed consent was obtained from all participants when they were weighed and measured.

### Measures

#### Demographics

Participants reported their age, sex, level of education (primary/secondary; high school/technical diploma; university), employment status (paid employment; unemployed or unpaid work; retired; student), and marital status (single; married or cohabiting; widowed or divorced).

#### The Adult Eating Behaviour Questionnaire (AEBQ)

The AEBQ [15] is a self-report measure of appetitive traits containing 35 items, each with 5 response options on a 5-point Likert scale (1 = “strongly disagree” to 5 = “strongly agree”). It is divided into two groups of subscales, four ‘food approach’ and four ‘food avoidance’ subscales. ‘Food-onset’ or ‘food approach’ subscales are comprised of: five Hunger items (e.g., ‘I often feel so hungry that I have to eat something right away’); four Food Responsiveness items (e.g., ‘I often feel hungry when I am with someone who is eating’); five Emotional Over-Eating items (e.g., ‘I eat more when I’m anxious’); and three Enjoyment of Food items (e.g., ‘I enjoy eating’). The four ‘food avoidance’ or ‘eating-offset’ subscales include: four Satiety Responsiveness items (e.g., ‘I cannot eat a meal if I have had a snack just before’); five Emotional Under-Eating items (e.g., ‘I eat less when I'm annoyed’); five Food Fussiness items (e.g., ‘I refuse new foods at first’); and four Slowness in Eating items (e.g., ‘I am often last at finishing a meal’). Subscale scores were calculated using the mean of the items for each scale.

#### Anthropometry

Participants were weighed and measured by the researchers, using standardized equipment (Tanita weighing scales and Seca stadiometer). Height was measured in centimeters to the nearest cm and weight was measured in kilograms to the nearest 100 g. These measures were used to calculate BMI (kg/m^2^) and weight categories (healthy weight: BMI = 18.5–24.9; overweight: BMI = 25–29.9; obesity: BMI > 30) [34].

#### Currently trying to lose weight

Participants were asked if they were currently trying to lose weight (yes; no), as weight management could be associated with appetitive traits and BMI [28, 35].

### Statistical analyses

Data were checked for missing values and outliers for height weight or BMI, with cut-off points of 16–52 kg/m^2^, and each item’s skewness and kurtosis were examined. The percentage of missing data for paper format questionnaires ranged from 0.1 to 0.4%, from 27/35 items. The missing cases were imputed using the Expectation Maximization method [36]. Less than 0.5% of values were imputed for all items (factors).

Normal distribution of the constructs or latent factors and individual items was observed, except for two Enjoyment of Food items (items AEBQ-Q1 and AEBQ-Q3) with values > 1 for skewness and kurtosis. Values deviated from normal (− 1–1) for skewness (− 1.6) and for kurtosis (3.7), however, these have been accepted as a less stringent rule for the upper threshold of normality, with no values exceeding 3 for skewness and 10 for kurtosis [37]. CFA was carried out using SPSS AMOS version 24. CFA is a method that allows for the assessment of fit between observed data and a previously conceptualized, theoretically grounded model that specifies the proposed causal relations between latent factors and their observed (i.e., measurable) variables [38]. Goodness of fit was assessed using: the comparative fit index (CFI), the normed fit index (NFI) and the root mean square error of approximation (RMSEA). CFI and NFI values close to 0.90 and RMSEA values of less than 0.05, with the lower-bound confidence interval closest to zero (0) and the higher-bound confidence interval less than 0.08 are considered indications of good fit [39, 40]. However, the best model fit can also be assessed by the lowest absolute value of the AIC (Akaike’s Information Criteria) and the BIC (Bayesian Information Criterion) [40–43]. The most parsimonious (the data that can explain the simplest model) approach is to select the model when the magnitude of fit changes by at least two points in the AIC or BIC [41, 43] (Supplementary material 2).

Other statistical analyses were performed using SPSS version 24. The internal reliability of each scale was assessed using Cronbach’s alpha (a measure of internal consistency), and test–retest reliability (an assessment of the extent to which a measure varies from one use to another). Test–retest reliability was quantified using intra-class correlation coefficients (ICC) [44] using a two-way random method [45]. Values > 0.7 indicate good external reliability [42]. Pearson’s correlation coefficients were used to show the associations between appetitive traits. Simple associations between appetitive trait means and BMI were also examined using Pearson’s correlation coefficients. We ran a Spearman’s Rho for Enjoyment of Food because it was not normally distributed, but we report the Pearson’s correlations as results were the same. Multivariable linear regression analyses were used to test for associations between BMI (as the independent variable) and each appetitive trait (as the dependent variable), adjusting for sex, age, mode of questionnaire (sample: paper copy vs online), level of education, marital and employment status, given they could act as statistical confounders. These confounders were selected based on previous research [20] and multivariable linear regression analyses between appetitive traits and currently trying to lose weight, education and employment status reported in Supplementary material 3. No results are presented for sex and age as no significant results were found. Multivariable linear regression analyses to test for associations between eight AEBQ-Esp subscales and unadjusted and adjusted associations with BMI by those currently trying to lose weight (*n* = 564) or not (*n* = 455) were also examined.

## Results

### Translation and Think Aloud methodology

This sample comprised 11 adults between 19 and 70 years of age (44.9 ± 16.4), and included 6 males (1 primary school, 1 middle school, 2 high school, 1 bachelor’s degree and 1 master’s level of education participants) and 5 females (1 primary school, 3 middle school and 1 PhD level of education participants). Participants were asked to think out loud while they completed the AEBQ. All items were understood by participants and were therefore retained. The reading level of the questionnaire was judged to be above primary school level (> 12 years of age).

### Participants

Overall, 1341 (734 online format; 419 paper versions) participants provided informed consent and were weighed and measured. Of these, 1153/1341 (86%) participants remained after eliminating incomplete questionnaires (> 50% of data missing). A further 110 participants were eliminated due to incomplete AEBQ data (9.5%), 15 participants due to incomplete primary school education level (1.3%), and 5 participants (0.4%) due to acquiescence bias (the tendency to agree with all the questions asked) with responses as assessed through case analysis via visual scanning. The final sample (1023/1341 [76.2%]) was comprised of 621 (60.7%) females, with a mean age of 36.8 ± 12.8 years, and mean BMI of 26.1 ± 5) (Table 1). Excluded participants were younger (24.6 ± 5.6 years), male (61%) and had a mean BMI of 23.4 ± 3.1 kg/m^2^. The AEBQ-Esp was completed a second time (2 ± 0.32 weeks after the first reply) by a total of 88 participants (26 males; 62 females), aged 36.0 ± 12.0 years (18 to 67 years old), with a BMI of 26.0 ± 4.95 (Table 1).

**Table 1:** Socio demographic characteristics of participants

Variable	Total (*n* = 1023)	Test–retest (*n* = 88)
Age (years)		
Mean ± SD	36.8 ± 12.8 years	36.0 ± 12.0
18–29	233 (22.8%)	33 (37.5%)
30–59	676 (66.1%)	51 (58.0%)
60+	114 (11.1%)	4 (4.5%)
Sex		
Male	402 (39.3%)	26 (29.5%)
Female	621 (60.7%)	62 (70.5)
BMI categories		
Mean ± SD	26.1 ± 5	26.0 ± 5
Underweight	16 (1.6%)	–
Healthy weight	372 (36.4%)	47 (53.4%)
Overweight	381 (37.2%)	22 (25%)
Obese	254 (24.8%)	19 (21.6%)
Education		
Primary/secondary	282 (27.6%)	88 (100%)
High school/technical diploma	308 (30.1%)	
University	433 (42.3%)	
Employment		
Paid employment	689 (67.4%)	72 (81.8%)
Unemployed or unpaid work	164 (16.0%)	16 (18.2)
Retired	45 (4.4%)	
Student	124 (12.1%)	
Marital status		
Single	342 (33.4%)	44 (50%)
Married or cohabiting	583 (57.0%)	36 (40.9%)
Widowed or divorced	96 (9.4%)	8 (9.1%)
Currently trying to lose weight	*n* = 1019	
Yes	564 (55.3%)	54 (61.4%)
No	455 (44.7%)	34 (38.4%)

### Confirmatory factor analysis

Model 1, which included all 8 factors of the original AEBQ (35 items), resulted in a reasonable fit: RMSEA = 0.058, CFI = 0.0864 and NFI = 0.832 [46]. Considering that the previous Australian validation of the AEBQ resulted in a better model fit when the Hunger subscale was removed, CFA was tested using a seven-factor model eliminating Hunger (Model 2). Model 2 (30 items), resulted in a similar fit: RMSEA = 0.063, CFI = 0.0868 and NFI = 0.842. However, Model 2 revealed smaller AIC and BIC values than Model 1, giving a meaningful magnitude of change of > two points, suggesting a better model fit (Table 2).

**Table 2:** Confirmatory Factor Analysis for 1023 participants who completed the AEBQ; and compared to the original AEBQ paper

	Model 1 (eight-factors)	Model 2 (seven-factors without Hunger)	Original AEBQ (eight-factors)	References*
RMSEA 90% CI	0.058 (0.056–0.061)	0.063 (0.060–0.066)	0.058 (0.056-0.061)	< 0.06
CFI	0.864	0.868	0.896	> 0.90
NFI	0.832	0.842	0.870	> 0.90
AIC	2580.770	2118.586	2613.345**	Smaller values, where the magnitude changes by at least 2 points***
BIC	3063.958	2512.956	3055.665**	

*RMSEA* root mean square error of approximation, *CFI* comparative fit index, *NFI* normed fit index, *AIC* Akaike’s Information Criteria, *BIC* Bayesian Information Criterion

*Hu and Bentler 1999 [46]; Dugard et al. 2010 [41]

**These AIC and BIC values cannot be used to compare against the AIC and BIC values from analyses in the original AEBQ paper with the values for the analyses on the AEBQ-Esp; they can only be used to test nested models within the same sample (e.g., Model 1 and Model 2 for the AEBQ-Esp)

***Burnham and Anderson 2003 [43]

### Descriptive statistics and internal and test–retest (external) reliability

Descriptive statistics (mean ± SD), internal validity (Cronbach’s *α*) and test–retest reliabilities (ICCs) for the eight factor AEBQ-Esp, and for the original eight-factor AEBQ validation study, are shown in Table 3. Results from the internal reliability of the AEBQ-Esp demonstrated all Cronbach’s alphas to be greater than 0.70, showing good internal consistency of the questionnaire. Test–retest reliability showed the AEBQ-Esp had higher than 0.70 reliability values for all subscales (0.70–0.91), showing good external reliability (Table 3). For a full set of AEBQ and AEBQ-Esp items (Supplementary material 4).

**Table 3:** Descriptive statistics and internal (*n* = 1023) and test–retest (*n* = 88) reliabilities for the eight factor AEBQ-Esp and the original AEBQ validation (*n* = 954)

Appetitive trait	AEBQ-Esp (present study)				AEBQ (Hunot 2016) [26]			
	Mean	SD	Internal reliability	Test-retest reliability 95% CI	Mean	SD	Internal reliability	Test-retest reliability 95% CI*
Hunger	2.87	0.76	0.70 (0.67, 0.73)	0.90 (0.85, 0.94)	2.92	0.78	0.75 (0.73, 0.78)	0.82 (0.73, 0.88)
Food Responsiveness	2.72	0.76	0.74 (0.71, 0.76)	0.90 (0.85,0.94)	2.98	0.78	0.75 (0.73, 0.78)	0.87 (0.81, 0.91)
Emotional Over-Eating	2.54	0.87	0.86 (0.84, 0.87)	0.91 (0.86,0.94)	2.74	0.98	0.90 (0.89, 0.91)	0.73 (0.60, 0.82)
Enjoyment of Food	3.98	0.74	0.78 (0.75, 0.80)	0.88 (0.82, 0.92)	4.00	0.74	0.86 (0.84, 0.87)	0.86 (0.79, 0.91)
Satiety Responsiveness	2.48	0.73	0.70 (0.66, 0.72)	0.87 (0.80,0.92)	2.61	0.81	0.75 (0.73, 0.78)	0.87 (0.80, 0.91)
Emotional Under-Eating	2.79	0.88	0.84 (0.83, 0.86)	0.78 (0.66, 0.86)	2.83	0.92	0.90 (0.89, 0.91)	0.77 (66, 0.85)
Food Fussiness	2.42	0.71	0.73 (0.71, 0.76)	0.70 (0.51, 0.79)	2.29	0.84	0.88 (0.86, 0.89)	0.91 (0.86, 0.94)
Slowness in Eating	2.69	0.93	0.82 (0.80, 0.84)	0.91 (0.86, 0.94)	2.62	0.97	0.88 (0.87, 0.90)	0.91 (0.86, 0.94)

*CI* confidence intervals

*Hunot 2016 [26]

### Associations between appetitive traits and with BMI

Table 4 shows the correlations between subscales. As expected, the ‘food approach’ subscales were positively inter-correlated and were generally negatively correlated with the ‘food avoidance’ subscales (Table 4), except for Hunger. Emotional Under-Eating was unexpectedly positively correlated to Hunger, Food Responsiveness and Emotional Over-Eating. The ‘food avoidance’ subscales were also positively inter-correlated however Food Fussiness was not significantly related Slowness in Eating (Table 4).

**Table 4:** Pearson’s correlations and multivariable regression analyses between the eight AEBQ-Esp subscales (*n* = 998) and unadjusted and adjusted correlations with BMI in a Mexican sample

	H	FR	EOE	EF	SR	EUE	FF	SE	BMI		
									Un-adjusted (*r*)	Un-adjusted^a^ (*β*)^&^ 95% CI	Adjusted^a^ (*β*)^&^ 95% CI
Food approach subscales											
Hunger	1	0.56**	0.45**	0.27**	0.06	0.19**	0.02	− 0.02	− 0.06	− 0.42 (− 0.85, 0.00)	− 0.09 (− 0.47,0.29)
Food Responsiveness		1	0.48**	0.40**	− 0.13**	0.11**	− 0.05	− 0.10**	− 0.04	− 0.30 (− 0.72, 0.13)	0.09 (− 0.29, 0.49)
Emotional Over-Eating			1	0.12**	0.03	0.01	0.09**	− 0.11**	0.14**	0.84** (0.47, 1.21)	0.94** (0.62, 1.27)
Enjoyment of Food				1	− 0.28**	− 0.02	− 0.28**	− 0.06	− 0.10**	− 0.69** (− 1.13, − 0.25)	− 0.06 (− 0.46, 0.34)
Food Avoidance Subscales											
Satiety Responsiveness					1	0.31**	0.18**	0.32**	− 0.11**	− 0.80** (− 1.24, − 0.35)	− 0.61** (− 1.01, − 0.21)
Emotional Under-Eating						1	0.09**	0.09**	− 0.10**	− 0.60** (− 0.97, − 0.24)	− 0.29 (− 0.61, − 0.04)
Food Fussiness							1	0.01	0.09**	0.65** (0.20, 1.01)	0.07 (− 0.34, 0.47)
Slowness in Eating								1	− 0.15**	− 0.84** (− 1.19, − 0.49)	− 0.70** (− 1.01, − 0.39)

*CI* confidence intervals

^a^Adjusted for age, sex, sample, education, marital and employment status

*Correlation is significant at the 0.05 level (2-tailed)

**Correlation is significant at the 0.01 level (2-tailed)

^&^*β* (beta) values are unstandarised

Three different models are presented for the associations with BMI: (i) unadjusted associations (Pearson’s correlations); (ii) unadjusted multivariable regressions (iii) multivariable regressions adjusted for sex, age, mode of questionnaire (sample: online vs paper copy), level of education, marital and employment status (Table 4). Emotional Over-Eating was associated, as expected, with a higher BMI [*β* = 0.94; (0.62–1.30)], but Hunger, Food Responsiveness and Enjoyment of Food were not. Satiety Responsiveness [*β* = − 0.61; (− 1.01–− 0.21)] and Slowness in Eating [β = − 0.70; (− 1.01–− 0.39)] were associated with a lower BMI, but not Food Fussiness or Emotional Under-Eating. Therefore, for those with a 1-unit higher in Emotional Over-Eating scores, and a 1-unit lower in Satiety Responsiveness and Slowness in Eating scores, a 0.94, 0.61 and 0.70 kg/m^2^ higher BMI was observed. Table 5 shows the multivariable regressions between the eight AEBQ-Esp subscales and unadjusted and adjusted (for age, sex, sample, education, marital and employment status) associated with BMI by those currently trying to lose weight (*n* = 564) or not (*n* = 455). After adjustment, Emotional Over-Eating was positively associated with BMI in those who were currently trying to lose weight [*β* = 0.63 (− 0.23, 1.04)]. Satiety Responsiveness was negatively associated with BMI in those who were currently trying to lose weight [*β* = − 0.53 (− 1.06, − 0.00)]. Among those not currently trying to lose weight, BMI was positively associated to Emotional Over-Eating [*β* = 0.43 (− 0.01, 0.85)] and negatively associated to Enjoyment of Food [*β* = − 0.63 (− 1.08, − 0.17)] and Slowness in Eating [*β* = − 0.51 (− 0.88, 0.15)], after adjustment. Therefore, for those who were currently trying to lose weight with a 1-unit higher in Emotional Over-Eating scores, and a 1-unit lower in Satiety Responsiveness and Slowness in Eating scores, a 0.63 and 0.53 kg/m^2^ higher BMI was observed. For those who were not currently trying to lose weight with a 1-unit higher in Emotional Over-Eating scores, and a 1-unit lower in Enjoyment of Food and Satiety Responsiveness, a 0.43, 0.63 and 0.51 kg/m^2^ higher BMI was observed.

**Table 5:** Multivariable regression analyses between the eight AEBQ-Esp subscales and unadjusted and adjusted correlations with BMI by those currently trying to lose weight (*n* = 564) or not (*n* = 455) in a Mexican sample

	Currently trying to lose weight (yes) Un-adjusted^a^ (*β*)^&^ 95% CI	Currently trying to lose weight (no) Un-adjusted^a^ (*β*)^&^ 95% CI	Currently trying to lose weight (yes) Adjusted^a^ (*β*)^&^ 95% CI^a^	Currently trying to lose weight (no) Adjusted^a^ (*β*)^&^ 95% CI^a^
Food approach subscales				
Hunger	− 0.21 (− 0.78, 0.36)	− 0.88** (− 1.34, − 0.37)	− 0.13 (− 0.47, 0.63)	− 0.40 (− 0.84, 0.05)
Food responsiveness	− 0.20 (− 0.76, 0.36)	− 0.78** (− 1.30, 0.27)	− 0.24 (− 0.26, 0.73)	− 0.28 (− 0.73, 0.17)
Emotional over-eating	0.39 (− 0.09, 0.87)	0.21 (− 0.29, 0.71)	0.63** (− 0.23, 1.04)	0.43* (− 0.01, 0.85)
Enjoyment of food	− 0.18 (− 0.77, 0.41)	− 1.18** (− 1.70, − 0.67)	0.45 (− 0.08, 0.97)	− 0.63** (− 1.08, − 0.17)
Food Avoidance subscales				
Satiety responsiveness	− 0.84** (− 1.44, − 0.25)	− 0.48 (− 1.02, 0.05)	− 0.53* (− 1.06, − 0.00)	− 0.31 (− 0.77, 0.15)
Emotional under-eating	− 0.51* (− 1.01, − 0.02)	− 0.53* (− 0.97, − 0.09)	− 0.14 (− 0.57, 0.30)	− 0.24 (− 0.62, 0.14)
Food fussiness	0.35 (− 0.27, 0.96)	0.56* (0.03, 1.08)	− 0.29 (− 0.83, 0.25)	0.16 (− 0.29, 0.62)
Slowness in eating	− 0.42 (− 0.89, 0.04)	− 0.71** (− 1.13, − 0.28)	− 0.34 (− 0.75, 0.07)	− 0.51** (− 0.88, 0.15)

^a^Adjusted for age, sex, sample, education, marital and employment status

^&^*β*(beta) values are unstandarised

*Correlation is significant at the 0.05 level (two-tailed)

**Correlation is significant at the 0.01 level (two-tailed)

## Discussion

This is the first study to examine the validity of the AEBQ in a large sample of Spanish speaking adults ranging from 18 to 80 years of age. The findings from this study show that the AEBQ-Esp is a valid and reliable tool for the measurement of appetitive traits in a Mexican population, in Spanish. CFA confirmed the use of a 7-factor model which eliminated the Hunger subscale from the original AEBQ, to produce a 30-item questionnaire that includes 3 ‘food approach’ traits (Food Responsiveness, Emotional Over-Eating and Enjoyment of Food) and 4 ‘food avoidance’ traits (Satiety Responsiveness, Emotional Under-Eating, Food Fussiness and Slowness in Eating). Internal validity and test–retest reliability estimates higher than 0.70 provide further support for the external reliability value of the questionnaire.

Positive intercorrelations between ‘food approach’ subscales were found and these ‘food approach’ subscales were generally negatively correlated with the ‘food avoidance’ subscales. However, Emotional Under-Eating was positively correlated with Hunger, and unexpectedly positively correlated to Food Responsiveness and Emotional Over-Eating. The ‘food avoidance’ subscales were also positively inter-correlated, with the exception of Food Fussiness, which was not significantly related to Slowness in Eating. This suggests the AEBQ-Esp subscales behave, for the most part, as would be expected, and in line with previous studies [15, 19–22, 30].

The study also provides further evidence for associations between some of the appetitive traits and BMI; higher Emotional Over-Eating was associated with a higher BMI, whereas higher Satiety Responsiveness and Slowness in Eating were associated with a lower BMI. However, no positive associations were found for Food Responsiveness and Enjoyment of Food, and no negative associations with Emotional Under-Eating and Food Fussiness; even after adjustment for sex, age, type of sample level of education, marital and employment status. These results are in line with the Australian validations of the AEBQ, except we did not find any associations with Food Fussiness (*β* = − 0.11, *p* < 0.001) [20, 21]. In the Chinese validation of the AEBQ, He et al., did not appear to find correlations between BMI and the ‘food approach’ subscales, but did find negative correlations with Satiety Responsiveness (*r* = − 0.18; *p* < 0.01), Food Fussiness (*r* = − 0.08; *p* < 0.01) and Slowness in Eating (*r* = − 0.16; *p* < 0.01) [21]. A recent Bulgarian validation of the 30-item AEBQ excluding the Hunger subscale, found positive correlations between BMI and Emotional Over-eating (*r* = 0.19; *p* < 0.01), and negative correlations between BMI and both Satiety Responsiveness (*r* = − 0.14; *p* < 0.01) and Emotional Under-eating (*r* = − 0.21; *p* < 0.01) [22]. Associations with BMI in this study, as in the original AEBQ development paper and the Australian, Bulgarian and Chinese validations, are small yet show that AEBQ-measured appetitive traits are associated with BMI across different populations.

This study is the first to show the effect of potential weight management efforts on the associations between the appetitive traits and BMI. Lower Satiety Responsiveness was associated with a higher BMI in those currently trying to lose weight. Lower Enjoyment of Food and Slowness in Eating were negatively associated with BMI in those not currently trying to lose weight. It is possible that ‘currently trying to lose weight may be acting as a potential moderator of the relationship between appetitive traits and BMI [27, 28]. Detailed investigation of this relationship needs to be explored in future research studies.

CFA tested two versions of the AEBQ; one with eight factors including Hunger and the other a seven-factor model eliminating Hunger. It revealed that a seven-factor structure without the Hunger subscale was a better model fit. The literature suggests that when the model fit ranked according to the AIC and BIC differs by at least two points, as the results show in this study, the model with the lowest values should be retained [43]. The exclusion of Hunger was also suggested in the original AEBQ development paper when no associations between Hunger and weight were found [15] and supported by the Australian CFA validation of the AEBQ [20] and the adolescent validation of the AEBQ [30]. Similarly, validation of the AEBQ in an adolescent sample confirmed a seven-factor model excluding Hunger was a better model fit [30]. However, the Chinese validation found that the eight-factor model including Hunger was a better model fit, when compared to a seven-factor model which included Food Responsiveness and Hunger items loaded onto one subscale [21]. They did not however use a CFA to assess a model without the Hunger subscale, which would be recommended. Results from this study provide further evidence that the Hunger items included in the original AEBQ should perhaps be excluded from future studies using the AEBQ or AEBQ-Esp. The Hunger items potentially relate to internal ‘states’ rather than a trait. These items may therefore be more affected by temporal factors such as the time of the last meal [29, 47]. Internal states are partly driven by ‘episodic’ signals (i.e., a pattern of food episodes), involved in meal-to-meal variation in appetite. In contrast, traits produce a more ‘tonic’ regulation of appetite that relates to longer term energy reserves and is more stable or trait-like [29]. Thus, we suggest Hunger may not be a trait but rather, an internal state. Studies considering inclusion of this subscale should also consider the findings from the Australian study, which found a negative association between Hunger and weight, which was unexpected (*r* = − 0.13; *p* < 0.01) [20].

Results from this study, as in the original AEBQ development paper and the Australian, Bulgarian and Chinese validations are modest, yet show that AEBQ-measured appetitive traits are consistently associated with BMI across different populations, after adjusting for sex, age, mode of questionnaire (online vs paper copy) and whether participants were trying to lose weight. However, only three appetitive traits were associated with BMI in the present study; Emotional Over-Eating, Satiety Responsiveness and Slowness in Eating. This finding is similar to the Australian validation [20] and the Chinese validation [21] that showed fewer associations with BMI than the original AEBQ as well as those found in children [9, 11, 12]; although Mallan et al., did find negative associations with Food Fussiness, as did He et al., and unexpectedly with Hunger. In contrast to the previous studies, objective measures of height and weight were obtained in this study, and we controlled for whether someone was actively trying to control their weight. Future research should also look at the potential associations between individual appetitive traits and BMI over time. Studies should seek to unpick further the relationships between the traits themselves and explore how these relationships influence any observed associations with BMI.

The lack of a relationship between Food Responsiveness and BMI is particularly surprising, especially given the consistent relationship between this trait and adiposity within pediatric samples [14, 15]. Mexico is considered to be a highly obesogenic environment [48], which could lead Mexican adults to be poor at recognizing this trait in themselves. It could also reflect a cultural tendency towards overeating, leading to a lack of awareness around whether the person is responding to the external food environment. There was also no association found with Enjoyment of Food and BMI, again in contrast to studies with children. This could be a reflection of the self-report nature of the AEBQ versus the parent-report CEBQ; people who are affected by overweight may feel that they would rather not admit that they enjoy food; parents, on the other hand, may feel less concerned about describing their child’s enjoyment of food and mealtimes. Longitudinal studies are needed to assess the direction of the relationship between appetitive traits and weight. The inclusion of better measures of restraint, to control for this potential confounder might also enhance our understanding of why these different relationships are seen in children and adults.

The null association between Food Fussiness and BMI is less surprising and provides further evidence that the relationship between fussy eating and weight in adults is unclear; inconsistent relationships with weight are also observed among children when using the CEBQ [9, 12, 49]. Fussy eating has been associated with a series of anomalous eating behaviors and attitudes towards food, particularly rejecting food based on sensory and olfactory characteristics, and contact with other food or touch by another person [50]. However, eating a diet that is low in dietary variety does not necessarily mean that the diet is low in energy, and may therefore not affect weight. For example, it is not uncommon for children to be fussy with vegetables and protein foods, but unselective with more energy dense foods.

### Limitations

The cross-sectional nature of the study is an important limitation. No causal inferences can be made from the associations between the AEBQ-Esp subscales and BMI observed in the current study. Prospective longitudinal research is required to understand the directionality of associations in this population, as has been done in children [51]. Adiposity may also influence the expression of appetitive traits, and the relative strength of the direction of the associations may vary over the life course. The use of CFA to examine the validity of the AEBQ-Esp, could have led to overgeneralized cut-off values [52], but it is considered the best method to test the construct validity of a questionnaire developed through Principal Component Analysis or Exploratory Factor Analysis [53]. A further limitation may have resulted from the recruitment strategy. Participants were recruited from a local dental clinic perhaps indicative that this population may have been suffering of dental issues which can affect their eating. This may limit the generalizability of the findings. Additionally, our sample was an opportunity sample and we did not collect information on the number of potential participants approached who chose not to participate in the study, nor their characteristics and thus may not be representative of the general population of adults in Guadalajara. Moreover, this is the first study to examine the validation of the AEBQ in Spanish but is limited to a Spanish-speaking population in Mexico. The AEBQ-Esp should be validated for use in different indigenous languages around Mexico, and in other Spanish-speaking regions. However, studies to date do suggest the structure of the AEBQ is relatively stable. Estimates of overweight and obesity in the whole sample were slightly lower than those found overall in Mexico [62% (AEBQ-Esp sample) versus 72.5% (Mexico)] [54]. Results from the Think-Aloud interviews used in this study suggest a primary school education is required to understand the questionnaire. Caution is therefore advised when using the questionnaire as a self-report method of eating assessment in participants with low readability, as they may not understand the intention of the questions. Further studies should also be carried out to observe population differences between AEBQ-Esp subscales and BMI during early and older adulthood.

## Conclusions

The AEBQ-Esp will enable population level data collection of a range of appetitive traits that are of interest in the aetiology of weight variation and, in particular, obesity risk. The findings from this study suggest that associations with BMI may be fewer and smaller than expected, and there is a need to explore these associations further across the lifespan in other Spanish-speaking populations.

## What is already known on this subject?

Appetitive traits are associated with weight in children and adults. The Adult Eating Behaviour Questionnaire (AEBQ) is a valid and reliable psychometric measure of appetitive traits, that has been validated in several countries but not in Mexico in Spanish; a country with a highly obesogenic environment.

## What does this study add?

The AEBQ-Esp is a valid and reliable tool for the measurement of appetitive traits in a Mexican population, in Spanish. In this population, higher Emotional Over-Eating was associated with a higher BMI, and higher Satiety Responsiveness and Slowness in Eating were associated with a lower BMI.

## Supplementary Information

Below is the link to the electronic supplementary material.

Supplementary file1 (DOCX 21 KB)

Supplementary file2 (DOCX 20 KB)

Supplementary file3 (DOCX 20 KB)

Supplementary file4 (DOCX 20 KB)

## Data Availability

The datasets used and/or analysed during the current study are available from the corresponding author upon reasonable request.

## Abbreviations

AEBQ: Adult Eating Behaviour Questionnaire
AIC: Akaike’s Information Criteria
BIC: Bayesian Information Criterion
CEBQ: Child Eating Behaviour Questionnaire
CFA: Confirmatory Factor Analysis
CFI: Comparative fit index
ICC: Intra-class correlation coefficients
NFI: Normed fit index
RMSEA: Root mean square error of approximation
