# Supplementary material for: Examining the validity and consistency of the Adult Eating Behaviour Questionnaire-Español (AEBQ-Esp) and its relationship to BMI in a Mexican population
Source: Eat Weight Disord. 2021 May 8;27(2):651–63. doi: 10.1007/s40519-021-01201-9 (PMC8933343; doi:10.1007/s40519-021-01201-9)
Supplement: Supplementary file 1 — Supplementary file1 (DOCX 21 KB) [file 40519_2021_1201_MOESM1_ESM.docx]

**Examining the validity and consistency of the Adult Eating Behaviour Questionnaire – Español (AEBQ-Esp) and its relationship to BMI in a Mexican population**

**Eating and Weight Disorders - Studies on Anorexia, Bulimia and Obesity**

Hunot-Alexander, C., Arellano-Gómez, L., Smith, A., Kaufer-Horwitz, M., Vasquez-Garibay, E. M., Romero-Velarde, E., Fildes, A., Croker, H., Llewellyn, C., Beeken, R. J.

Correspondence: Dr. Rebecca Beeken. Yorkshire Cancer Research University Academic Fellow, Leeds Institute of Health Sciences, Level 10, Worsley Building, Clarendon Way, Leeds, LS2 9NL, United Kingdom. Orcid.org/0000-0001-8287-9351 [R.Beeken@leeds.ac.uk](mailto:R.Beeken@leeds.ac.uk)

**Supplementary material 1.** A brief description of the translation method used to examine the validation of the AEBQ-Esp.

For the purpose of translating the AEBQ into Spanish several procedures took place, in order to achieve the best translation of the questionnaire.

1. The original author of the AEBQ (CHA) who is fluent in English and has an English mother and born and lives in Mexico, able to understand the nuances of both languages, in particular British English and Mexican Spanish, performed the forward translation of the AEBQ into Spanish.
2. Four researchers fluent in both Spanish and English (LPAG, MKH, EMVG, ERV) also discussed the translation of the final questionnaire and made minor adjustments to various words.
3. Once a final translated version was obtained, the principal researcher (CHA) carried out cognitive interviews, using the Think Aloud method [1], similarly to that explained in the AEBQ validation paper [2]. Participants included six males ages 19 to 70 (43.5±18.9), one with primary school education, two high school education levels, one with a bachelor´s degree and two master´s level education; five females (46.6±14.8) were interviewed, one with primary level education, one a bachelor´s degree and two master´s level education.
4. Think aloud techniques were used, where participants were asked to ‘read out loud’ instructions and items of the translated questionnaire and say ‘out loud’ what their answers were. Paraphrasing techniques were used, such as repeating the same phrase they had just read out loud to see if they had understood the questions. Probes on what they had answered out loud, were also used, mentioning possible answers to the response options available for them to confirm. This method allows to assess the understanding of the questionnaire.
5. On top of this, participants understood the questionnaire and a Flesch Reading Ease assessment gave the AEBQ an easy to read score (82.3) [3].
6. The backward translation was then given to a certified translator, who although Mexican-American, is married to a British Citizen and thus British nuances in language were also able to be discussed.
7. The final translation of the AEBQ was then obtained.

References

1. Fox MC, Ericsson KA, Best R (2011) Do procedures for verbal reporting of thinking have to be reactive? A meta-analysis and recommendations for best reporting methods. Psychol Bull 137:316–344 . doi: 10.1037/a0021663

2. Hunot C, Beeken RJ, Goodman W, Fildes A, Croker H, Llewellyn C, Steinsbekk S (2019) Confirmation of the Factor Structure and Reliability of the ‘Adult Eating Behavior Questionnaire’ in an Adolescent Sample. Front. Psychol. 10:1–10

3. (2018) Readability Formulas. In: Text Readability Consens. Calc. http://www.readabilityformulas.com/freetests/six-readability-formulas.php. Accessed 20 Jan 2020
