# Supplementary material for: Examining the validity and consistency of the Adult Eating Behaviour Questionnaire-Español (AEBQ-Esp) and its relationship to BMI in a Mexican population
Source: Eat Weight Disord. 2021 May 8;27(2):651–63. doi: 10.1007/s40519-021-01201-9 (PMC8933343; doi:10.1007/s40519-021-01201-9)
Supplement: Supplementary file 2 — Supplementary file2 (DOCX 20 KB) [file 40519_2021_1201_MOESM2_ESM.docx]

**Examining the validity and consistency of the Adult Eating Behaviour Questionnaire – Español (AEBQ-Esp) and its relationship to BMI in a Mexican population**

**Eating and Weight Disorders - Studies on Anorexia, Bulimia and Obesity**

Hunot-Alexander, C., Arellano-Gómez, L., Smith, A., Kaufer-Horwitz, M., Vasquez-Garibay, E. M., Romero-Velarde, E., Fildes, A., Croker, H., Llewellyn, C., Beeken, R. J.

Correspondence: Dr. Rebecca Beeken. Yorkshire Cancer Research University Academic Fellow, Leeds Institute of Health Sciences, Level 10, Worsley Building, Clarendon Way, Leeds, LS2 9NL, United Kingdom. Orcid.org/0000-0001-8287-9351 [R.Beeken@leeds.ac.uk](mailto:R.Beeken@leeds.ac.uk)

**Supplementary material 2.** Brief explanation of model fit indices for the validation of the AEBQ-Esp

In order to show whether the proposed model fits the data, correlations between the variables must be correctly accounted for [1]. It is recommended to consult several fit statistics when running a Confirmatory factor analysis (CFA), to assess whether they are consistent [2]. The normed fit index (NFI) indicates the degree to which the defined model improves fit over the null model; for example, a NFI of 0.90 means the defined model improves the fit by 90% relative to the null model [3]. A comparative fit index (CFI) of 0.90 to 0.95 suggests a good model fit, as does a Root-Mean-Square Error Approximation (RMSEA) ≥0.06 that it assesses how far a hypothesized model is from a perfect model [2, 3]. The Chi-square test is a measure of the difference between observed and expected [covariance matrices](https://en.wikipedia.org/wiki/Covariance_matrix) and should be non-significant. However, the Chi-square test readily reaches significance with large sample sizes even when all other indices indicate a good fit [1]. Factor loadings, which tell us about the relative contribution that a particular item makes to a factor [4], should be greater than 0.40 [5]. Out of several competing models, the model with the lowest AIC (Akaike’s Information Criteria) and BIC (Bayesian Information Criterion) values is considered the best fit to the data [1, 4]. The AIC and BIC are used as model selection criteria in this study. Given the AIC is a model statistic which penalizes a model for having a greater amount of variables by giving it a higher score, the lowest values for AIC was used to represent the best model fit [4].

References

1. Dugard P, Todman J, Staines H (2010) Factor analysis. In: Approaching multivariate analysis, 2nd ed. Routledge, London and New York, pp 177–205

2. Thompson B (1951) Exploratory and confirmatory factor analysis: Understanding concepts and applications, 1st c2004. American Psychological Association, Washington DC

3. Hu L, Bentler PM (1999) Cutoff criteria for fit indexes in covariance structure analysis: Conventional criteria versus new alternatives. Struct Equ Model 6:1–55 . doi: 10.1080/10705519909540118

4. Field A (2013) Discovering statistics using SPSS., 4th ed. SAGE Publications Ltd., London

5. Stevens JP (2009) Applied multivariate statistics for social sciences., 5th ed. Routledge, Hove, East Sussex
