# Supplementary material for: Examining the validity and consistency of the Adult Eating Behaviour Questionnaire-Español (AEBQ-Esp) and its relationship to BMI in a Mexican population
Source: Eat Weight Disord. 2021 May 8;27(2):651–63. doi: 10.1007/s40519-021-01201-9 (PMC8933343; doi:10.1007/s40519-021-01201-9)
Supplement: Supplementary file 3 — Supplementary file3 (DOCX 20 KB) [file 40519_2021_1201_MOESM3_ESM.docx]

**Examining the validity and consistency of the Adult Eating Behaviour Questionnaire – Español (AEBQ-Esp) and its relationship to BMI in a Mexican population**

**Eating and Weight Disorders - Studies on Anorexia, Bulimia and Obesity**

Hunot-Alexander, C., Arellano-Gómez, L., Smith, A., Kaufer-Horwitz, M., Vasquez-Garibay, E. M., Romero-Velarde, E., Fildes, A., Croker, H., Llewellyn, C., Beeken, R. J.

Correspondence: Dr. Rebecca Beeken. Yorkshire Cancer Research University Academic Fellow, Leeds Institute of Health Sciences, Level 10, Worsley Building, Clarendon Way, Leeds, LS2 9NL, United Kingdom. Orcid.org/0000-0001-8287-9351 [R.Beeken@leeds.ac.uk](mailto:R.Beeken@leeds.ac.uk)

**Supplementary material 3.**

**Multivariable regression analyses between the eight AEBQ subscales (N = 998) and** **currently trying to lose weight, education and employment status in a Mexican sample**

|  | **Currently trying to lose weight*** | | **Education**** | **Marital status †** | | **Employment status‡** |
| --- | --- | --- | --- | --- | --- | --- |
| **Food approach subscales** | |  | | |  | |
| Hunger | .23 (-.02, .06) | | .01 (-.06, .08) | **-.05* (-.10, -.00)** | | **.13** (.04, .21)** |
| Food responsiveness | -04 (-.01, .08) | | .29 (-.04, .10) | **-.09** (-.13, -.04)** | | **-.09** (-.13, -.04)** |
| Emotional over-eating | **.12** (.09, .16)** | | -00 (-.06, .05) | .01 (-.03, .06) | | -.01 (-.08, .06) |
| Enjoyment of food | -.01 (-.05, .02) | | **.18** (.11, .24)** | **-.09** (-.14, -.04)** | | .08 (-.00, .17) |
| **Food Avoidance subscales** | |  | | |  | |
| Satiety responsiveness | -.00 (-.07, .07) | | -.00 (-07, .07) | -.24 (-.08, .03) | | .08 (-.01, .16) |
| Emotional under-eating | -.02 (-.06, .01) | | **-.09** (.03, .14)** | -.04 (-.08, .00) | | -.07 (-.14, .01) |
| Food fussiness | **.04* (.00, .09)** | | **-.22** (-.29, -.15)** | .03 (-.03, .08) | | .02 (-.07, .10) |
| Slowness in eating | **-.06 **(-.10, -.03)** | | .01 (-.05, .06) | -.02 (-.06, .02) | | **.10** (.03, .16)** |

* Currently trying to lose weight (Yes, No)

** Education (Primary/Secondary; High school/Technical diploma; University)

† Marital status (single; married or cohabiting; widowed or divorced).

‡Employment status (paid employment; unemployed or unpaid work; retired; student)

*Correlation is significant at the 0.05 level (2-tailed).

**Correlation is significant at the 0.01 level (2-tailed).
