# Supplementary material for: Examining the validity and consistency of the Adult Eating Behaviour Questionnaire-Español (AEBQ-Esp) and its relationship to BMI in a Mexican population
Source: Eat Weight Disord. 2021 May 8;27(2):651–63. doi: 10.1007/s40519-021-01201-9 (PMC8933343; doi:10.1007/s40519-021-01201-9)
Supplement: Supplementary file 4 — Supplementary file4 (DOCX 20 KB) [file 40519_2021_1201_MOESM4_ESM.docx]

**Examining the validity and consistency of the Adult Eating Behaviour Questionnaire – Español (AEBQ-Esp) and its relationship to BMI in a Mexican population**

**Eating and Weight Disorders - Studies on Anorexia, Bulimia and Obesity**

Hunot-Alexander, C., Arellano-Gómez, L., Smith, A., Kaufer-Horwitz, M., Vasquez-Garibay, E. M., Romero-Velarde, E., Fildes, A., Croker, H., Llewellyn, C., Beeken, R. J.

Correspondence: Dr. Rebecca Beeken. Yorkshire Cancer Research University Academic Fellow, Leeds Institute of Health Sciences, Level 10, Worsley Building, Clarendon Way, Leeds, LS2 9NL, United Kingdom. Orcid.org/0000-0001-8287-9351 [R.Beeken@leeds.ac.uk](mailto:R.Beeken@leeds.ac.uk)

**Supplementary material 4.** Full set of AEBQ and AEBQ-Esp items.

| **AEBQ*** | **Subscales** | | **AEBQ-Esp**** |
| --- | --- | --- | --- |
| I often notice my stomach rumbling | Hunger | Hambre | Con frecuencia me doy cuenta que mi estómago hace ruidos de hambre |
| If I miss a meal I get irritable |  |  | Si me salto una comida, me pongo irritable |
| I often feel so hungry that I have to eat something right away |  |  | Con frecuencia siento tanta hambre que tengo que comer algo de inmediato |
| I often feel hungry |  |  | Con frecuencia siento hambre |
| If my meals are delayed I get light-headed |  |  | Si se retrasan mis comidas, me siento mareado (a) |
| I often feel hungry when I am with someone who is eating | Food Responsiveness | Respuesta a los Alimentos | Si estoy con alguien que está comiendo, frecuentemente me siento hambriento(a) |
| Given the choice, I would eat most of the time |  |  | Si tuviera la opción, comería la mayor parte del tiempo |
| I am always thinking about food |  |  | Siempre estoy pensando en comida |
| When I see or smell food that I like, it makes me want to eat |  |  | Cuando veo o huelo la comida que me gusta, me hace querer comer |
| I eat more when I'm annoyed | Emotional over-eating | Sobre-  Alimentación Emocional | Como más cuando estoy irritado(a) o enfadado(a) |
| I eat more when I'm worried |  |  | Como más cuando estoy preocupado(a) |
| I eat more when I'm upset |  |  | Como más cuando estoy molesto(a) |
| I eat more when I´m anxious |  |  | Como más cuando estoy ansioso(a) |
| I eat more when I'm angry |  |  | Como más cuando estoy enojado(a) |
| I love food | Enjoyment of Food | Disfrute de los Alimentos | Me encanta la comida |
| I enjoy eating |  |  | Disfruto comer |
| I look forward to mealtimes |  |  | Espero con ganas la hora de las comidas |
| I often leave food on my plate at the end of a meal | Satiety Responsiveness | Respuesta a la Saciedad | Con frecuencia dejo alimentos en mi plato al final de una comida |
| I often get full before my meal is finished |  |  | Con frecuencia me siento lleno(a) antes de que termine mi comida |
| I cannot eat a meal if I have had a snack just before |  |  | No puedo comerme mi comida si he comido un bocadillo justo antes |
| I get full up easily |  |  | Me siento lleno(a) muy fácilmente |
| I eat less when I'm worried | Emotional Under-Eating | Sub-  Alimentación Emocional | Como menos cuando estoy preocupado(a) |
| I eat less when I'm angry |  |  | Como menos cuando estoy enojado(a) |
| I eat less when I'm upset |  |  | Como menos cuando estoy molesto(a) |
| I eat less when I'm annoyed |  |  | Como menos cuando estoy irritado(a) o enfadado(a) |
| I eat less when I'm anxious |  |  | Como menos cuando estoy ansioso(a) |
| I often decide that I don’t like a food, before tasting it | Food Fussiness | Actitud remilgosa | Con frecuencia decido que no me gusta un alimento, antes de probarlo |
| I refuse new foods at first |  |  | Al principio rechazo alimentos nuevos |
| I enjoy tasting new foods |  |  | Me gusta probar alimentos nuevos |
| I am interested in tasting new food I haven’t tasted before |  |  | Me interesa probar alimentos nuevos que no he probado antes |
| I enjoy a wide variety of foods |  |  | Disfruto una gran variedad de alimentos |
| I am often last at finishing a meal | Slowness in Eating | Lentitud para comer | Con frecuencia soy el (la) último(a) en terminar mis alimentos |
| I eat more and more slowly during the course of a meal |  |  | Como cada vez más lento durante el transcurso de una comida |
| I eat slowly |  |  | Como despacio |
| I often finish my meals quickly |  |  | Con frecuencia termino mis alimentos rápidamente |

| Food Approach subscales; Sub-escalas pro-ingestión | Food Avoidance subscales; Sub-escalas anti-ingestión |
| --- | --- |

*Response option: Strongly disagree, Disagree, Neither agree nor disagree, Agree, Strongly agree.

**Opción de respuestas: Muy en desacuerdo, En desacuerdo, Ni en acuerdo ni en desacuerdo, En acuerdo, Muy en acuerdo
